# Quality of life and socioeconomic and educational status in patients with congenital hypothyroidism

**DOI:** 10.1038/s41390-024-03170-y

**Published:** 2024-04-02

**Authors:** Emmi Danner, Reijo Sund, Harri Sintonen, Laura Niuro, Harri Niinikoski, Hanna Huopio, Liisa A. Viikari, Jarmo Jääskeläinen

**Affiliations:** 1 Department of Pediatrics, University of Eastern Finland and Kuopio University Hospital, Kuopio, Finland; 2 School of Medicine, Institute of Clinical Medicine, University of Eastern Finland, Kuopio, Finland; 3 Department of Public Health, University of Helsinki, Helsinki, Finland; 4 Department of Pediatrics, University of Turku and Turku University Hospital, Turku, Finland

## Abstract

**Background:**

The aim of this study was to investigate the influence of primary congenital hypothyroidism (CH) on quality of life, level of education and socioeconomic status (SES).

**Methods:**

Two independent study cohorts, a national and a regional, were collected from Finnish national registers and patient records. Data on social security benefits, SES, marital status, and education were collected from Statistics Finland. Health-related quality of life (HRQoL) was studied in the regional patient cohort with the standardized 15D and 16D instruments.

**Results:**

There were no statistically significant differences in education level, marital status, or SES between CH patients (*n* = 40) and their matched controls at the age of 25 years. The mean 15D score was both statistically significantly and clinically importantly lower in CH patients (*n* = 29) than controls (0.904 vs. 0.953, *p* = 0.008). CH patients reported significantly lower scores across various dimensions of physical and mental HRQoL, including breathing, sleeping, speech, excretion, mental function, distress, and vitality. The mean 16D score was lower in CH patients compared to controls (0.917, vs. 0.947), but without statistical significance.

**Conclusion:**

SES of CH patients did not differ from matched controls. Thus, most CH patients integrate well into society, but their HRQoL is impaired.

**Impact:**

- Most patients with primary congenital hypothyroidism integrate well into society. In the current study, their socioeconomic and marital status did not differ from matched controls at the age of 25 years.
- However, health-related quality of life measured using 15D instrument was impaired.
- Every fourth patient reported that congenital hypothyroidism influenced everyday life.

## Introduction

Primary congenital hypothyroidism (CH) is the most frequent congenital endocrine disorder affecting one in 1000–3000 live births globally.^1–7^ Untreated CH may lead to severe intellectual disability, which can be prevented by newborn screening and prompt treatment. In countries with newborn screening programmes, most CH patients have normal neurodevelopmental outcome. Still, children and adults with CH may have cognitive and motor deficits and behavioral difficulties.^8–11^ In addition, associated malformations and chronic diseases may affect everyday life.^12,13^

Quality of life is an important endpoint in medical research. In CH, quality of life has mainly been studied in patients born in the first decade of the modern screening era, i.e. the 1980s.^14–16^ Thereafter, the treatment has improved by increasing the initial levothyroxine dose and younger onset age of therapy. The results of these earlier studies are contradictory. In a Japanese study including 43 patients, WHO/QOL-26 questionnaire was used to measure the health-related quality of life (HRQoL) of young adults with CH, and it did not differ from healthy controls.^14^ In the Netherlands, both children (*n* = 82) and young adults (*n* = 69) reported lower HRQoL than their healthy peers in age-specific TNO-AZL Questionnaires for Quality of Life.^15,17^ Furthermore, in a French study using SF-36 questionnaire, CH patients (*n* = 1162) had a statistically significant but not clinically important impairment of HRQoL compared to the general population.^16^

In addition to quality of life, socioeconomic status (SES) in adulthood is another important endpoint. In earlier studies, covering patients born in the 1980s, most CH patients have been found to integrate well into society.^16^ On the other hand, it has been reported that CH patients have delayed social development,^15^ they live longer with their parents and are less likely to attain the highest level of education compared to their healthy peers.^16^

In this study we investigated the level of education, need for special education, SES, employment, and marital status in a nationwide population-based cohort of Finnish CH patients and their matched controls. Furthermore, we investigated the health-related quality of life using 15D and 16D instruments in CH patients from the regional cohort compared to a Finnish reference population.

## Background

In Finland, a nationwide screening program for congenital hypothyroidism with complete coverage was implemented in 1980.^18^ During the 1980s, laboratory testing for CH was centralized in Finland. Since the early 1990s, the CH screening program has been multicentric, managed by central hospitals and regional laboratories. With coverage approaching 100%, nearly all children born in Finland undergo screening.^18,19^

Finland distinguishes itself from other Western countries by utilizing cord blood for screening instead of dry blood spot testing. Cord blood testing enables earlier confirmatory testing and initiation of levothyroxine treatment compared to countries employing dry blood spot screening. In Finland, the mean age at the start of the treatment is 4 days.^20^ If the TSH value exceeds the threshold, a confirmatory test (TSH and free thyroxine, fT4) is conducted after 72 h of age. The cut-off limits have either remained unchanged or have been lowered, depending on the methods used in different laboratories and clinical practices of hospitals. The cut-off limit for the screening test of primary CH has ranged between TSH 25–40 mU/l, and for the confirmatory test, between TSH 20–40 mU/l.

In contrast to most reports worldwide, the incidence of CH has remained constant in Finland over the last decades, with an incidence rate of 1:2 783.^6^

## Subjects

**Fig. 1:**
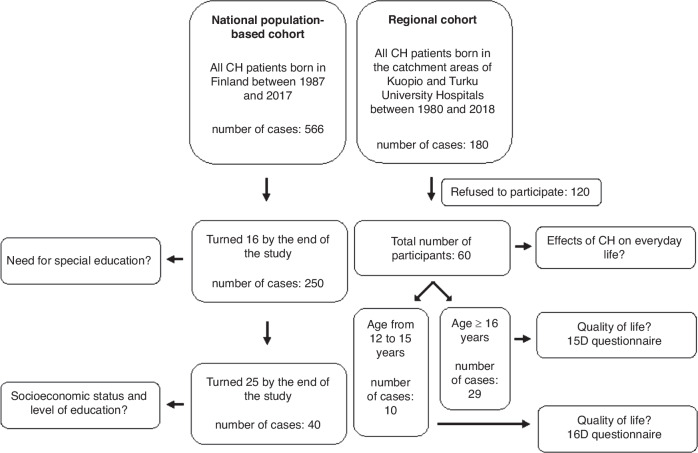
Flowchart illustrating two distinct study cohorts: a national cohort for a registration-based study and a regional cohort. The age limits and sizes of the study populations are indicated. The research questions for each study group are presented on the sides of the flowchart. CH congenital hypothyroidism.

We collected two separate study cohorts, a national and a regional (Fig. 1).

As there is no specific CH register in Finland, the nationwide cohort was identified from four distinct national population-based registers: The Prescription Register (established in 1993) maintained by The Social Insurance Institution of Finland, The Care Register for Health Care (established in 1994) (formerly the Hospital Discharge Register, with information available since 1969), The Medical Birth Register (established in 1987) and The Register of Congenital Malformations (established in 1963) maintained by The Finnish Institute for Health and Welfare. All permanent residents in Finland have a unique personal identity code, which makes these registers linkable. We studied patients born after the establishment of The Medical Birth Register (1987) to ensure full coverage.

The criteria to recognize CH patients from the aforementioned registers were a diagnosis of CH (ICD-10 E03.1 or E03.0 / ICD-9 243) under the age of two years (The Hospital Discharge Register and The Care Register for Health Care) and/or the purchase of levothyroxine (ATC H03AA01/03) under the age of one year (The Prescription Register), and/or the diagnosis of CH in The Medical Birth Register and/or The Register of Congenital Malformations. The inclusion criterion was a diagnosis of primary CH, which had to be recorded in one or more registers at least twice with at least two months’ interval, if not recorded as a cause of death. The purpose of these criteria was to exclude cases with suspected but unconfirmed CH, and to eliminate clear logging errors. The exclusion criteria were a diagnosis of end-stage renal disease (ICD10 N18/ICD9 585 or 586) or panhypopituitarism (ICD10 E23.00/ICD9 253.2), i.e., use of levothyroxine for an indication other than primary CH.

A matched control cohort was created using The Medical Birth Register. Two matched controls for each patient were randomly selected. The control subjects were matched for date of birth (+/−3 months), sex, mother’s age at childbirth (+/−2 years), number of fetuses, parity (1, 2, 3 or more) and the place of birth (hospital district).

The nationwide cohort was used to establish the SES and the level of education in adulthood. We studied patients and their controls who turned 25 years old at the latest in 2017. To evaluate the need for special education during comprehensive school, we studied patients who turned 16 years old at the latest in 2018.

To assess the quality of life, a regional cohort was collected from the catchment areas of Kuopio and Turku University Hospitals. Patients were identified by a diagnosis-based search from the patient records. The inclusion criteria were abnormal screening and confirmatory test results. In borderline cases, patients were included in the study, when a pediatric endocrinologist or a pediatrician diagnosed primary CH and levothyroxine was started before the age of 14 days. The fulfillment of the inclusion criteria was manually verified from the medical records by two of the authors (ED, LN). All patients aged 12 years or older, expect for one who had died, received the HRQoL questionnaire. Additionally, all patients and/or their parents received a questionnaire with an open question about the effects of CH on everyday life.

## Methods

For the national cohort, we collected information about social security benefits, SES, level of education, marital status, and number of children from Statistics Finland for patients 25 years of age or older in year 2017 and for their matched controls. The data were collected from the year when the subject turned 25. Information about special education was collected for patients and controls who turned 16 in 2018 at the latest (i.e. those who had completed comprehensive school). The data were linked and pseudonymized by Statistics Finland and provided to the researchers’ use via a remote access system.

The 15-dimensional HRQoL instrument for adults (15D)^21^ and the 16-dimensional instrument for adolescents (16D)^22^ are non-disease-specific multipurpose tools to evaluate a self-rated state of health covering emotional, physical, and social components. For the regional cohort, we used the standardized 15D instrument^21^ for patients aged 16 years or older. For 12- to 15-year-old patients, we used the 16D instrument.^22^ The dimensions of the 15D questionnaire include mobility, vision, hearing, breathing (shortness of breath), sleeping, eating, speech, excretion (bladder and bowel function), usual activities, mental function, discomfort and symptoms, depression, distress, vitality, and sexual activity. The 16D questionnaire includes the same dimensions with a few exceptions: usual activities are replaced by school and hobbies and sexual activity is replaced by appearance and friends. For each dimension, the participants answer on a scale of 1–5 (with 1 indicating the best and 5 the worst level) describing their current state of health. The single index score, 15D or 16D score, which represents the overall HRQoL on a 0-1 scale (1 = full health, 0 = being dead) and the dimension level values (1 = no problems on the dimension, 0 = being dead) were calculated by using a set of population-based preference or utility weights. A difference of ± 0.015 in the 15D score is clinically important^23^ and is assumed to be similar for the 16D score. The HRQoL scores of the CH patients were compared to a representative sample of the age- and sex-standardized general population (*n* = 816) regarding the 15D questionnaire^24^ and to the age- and sex-standardized general population from 4 schools in the Helsinki metropolitan area (*n* = 373) regarding the 16D questionnaire.^25^ Mean dimension level values are used to visualize 15D and 16D group-level profiles.

In a separate questionnaire we asked an open question from the patients. The question was: Does hypothyroidism affect your/your child’s health or daily life in any way? If yes, please describe how.

Patients in the regional cohort were initially contacted by letter, delivered via postal service. In the event of no response, subsequent contact attempts were made at intervals of 4 and 8 weeks.

The Finnish Institute for Health and Welfare, The Social Insurance Institution of Finland, and Statistics Finland granted permission for this study. The study protocol was approved by the Ethics Committee of the Northern Savo Hospital District. According to Finnish legislation, patient consent is not required for register-based studies. However, for the regional HRQoL study, informed consent was obtained from the patients and, if needed, their parents in accordance with the ethical principles stated in the Declaration of Helsinki.

## Statistical methods

The data were analyzed using the Statistical Package for Social Sciences (SPSS for Windows, Version 27, IBM Corp., Armonk, NY).

Statistical significances of the differences in the means between the cases and controls were tested with the independent samples *t* test. The Pearson’s Chi-square test was used to compare differences in categorical variables between the study groups. A *p* value < 0.05 was considered statistically significant.

## Results

Between 1987 and 2017, 1,843,502 children were born alive in Finland. We identified 566 patients with CH of whom 250 turned 16 by the end of the study. Characteristics of these patients and their matched controls are described in Table 1. Forty of them turned 25 years old (i.e., were born between 1987 and 1992) by the end of the year 2017. There were no differences in the socioeconomic or marital status between CH patients and their matched controls (Table 2) at the age of 25 years. Three CH patients (8.6%) and 14 controls (21%) had a higher tertiary level education, whereas secondary education was the highest level of education for 60% of CH patients and 47% of controls. These differences were not statistically significant. Both groups received social security benefits similarly. The need for special education was studied in subjects aged 16 years or older (born between 1987 and 2002) resulting in a population of 250 CH patients and 499 controls. 14% of CH patients and 10% of controls needed special education. This difference was not statistically significant either (Table 3).

**Table 1:** Characteristics of patients with congenital hypothyroidism (CH) born between 1987 and 2002 in Finland and their controls.

	CH patients born in Finland between 1987 and 2002 *n* = 250	Controls *n* = 499	*p* value
Sex (males:females)	90:160	179:320	N/A
Duration of the pregnancy, days from the due date, median (IQR)	2 (−7–10)	−1 (−7–6)	*p* = 0.006^a^
Born over 14 days post-term, *n* (%)	25 (10.0%)	26 (5.2%)	*p* = 0.014^b^
Born full-term	208 (83.2) %)	436 (87.4%)	*p* = 0.121^b^
Born preterm after 32 gestation weeks, *n* (%)	15 (6.0%)	35 (7.0%)	*p* = 0.600^b^
Born extremely preterm before 32 gestation weeks, *n*	<3	<3	
Born from a twin pregnancy, *n* (%)	10 (4.0%)	20 (4.0%)	N/A
Born under 2500 g, *n* (%)	18 (7.2%)	31 (6.2%)	*p* = 0.606^b^
Born between 2500 and 4500 g, *n* (%)	218 (87.2%)	452 (90.6%)	*p* = 0.155^b^
Born over 4500 g, *n* %	14 (5.6%)	16 (3.2%)	*p* = 0.115^b^
Mother’s age at birth, years, mean (95% CI)	29.1 (28.4–29.8)	29.1 (28.6–29.6)	N/A
Firstborn	98 (39.2%)	196 (39.3%)	N/A
Second-born	94 (37.6%)	188 (37.7%)	N/A
Third or more born	58 (23.2%)	115 (23.0%)	N/A

*N/A* not applicable, matched controls, *95% CI*   95% confidence interval, *IQR* interquartile range.

^a^Mann–Whitney U-test.

^b^Pearson Chi-square.

**Table 2:** Socioeconomic status, level of education, social security benefits and family details at the age of 25 years of patients with congenital hypothyroidism born between 1987 and 1992 in Finland and their matched controls.

	CH cases (40)	Controls (80)
Sex:		
Male	17 (43%)	34 (43%)
Female	23 (58%)	46 (58%)
Socioeconomic status:	*CH cases (38)*	*Controls (78)*
At work	22 (58%)	47 (60%)
Student	6 (16%)	14 (18%)
Retired^a^	4 (11%)	4 (5.1%)
Unemployed	3 (7.9%)	8 (10%)
Unknown	3 (7.9%)	5 (6.4%)
Highest level of education:	*CH cases (40)*	*Controls (80)*
Comprehensive school	5 (13%)	12 (15%)
Secondary education	21 (53%)	32 (40%)
Lower tertiary/bachelor	11 (28%)	22 (28%)
Higher tertiary/master	3 (7.5%)	14 (18%)
Marital status:	*CH cases (38)*	*Controls (78)*
Unmarried	33 (87%)	69 (88%)
Married/divorced	5 (13%)	9 (12%)
Having children:	*CH cases (40)*	*Controls (79)*
Yes	7 (18%)	15 (19%)
No	33 (83%)	64 (81%)
Social security benefits:	*CH cases (38)*	*Controls (78)*
All transfers received, mean (95% CI)^b^	4837 € (2758–6917)	4295 € (3153–5438)
Social assistance, number of cases	4 (11%)	10 (13%)
Social assistance (all), mean (95% CI)^b^	245 € (0–508)	273 € (86–460)
Social assistance (if received), mean (95% CI)^b^	2328 € (415–4242)	2126 € (1255–2997)

The Pearson’s Chi-square test was used to compare differences between the study groups.

*P* value > 0.1 in all comparisons between the groups.

^a^Including disability retirement.

^b^Statistical significances of the differences in the means were tested with the independent samples t test.

**Table 3:** Need for special education at any year during comprehensive school in patients with congenital hypothyroidism and their matched controls born between 1987 and 2002 in Finland.

Need for special education	CH cases (250)	Controls (499)	*p* value^a^
Yes	35 (14%)	51 (10%)	*p* = 0.061
No	190 (76%)	430 (86%)	
Information not available	25 (10%)	18 (3.6%)	

^a^Pearson’s Chi-square test.

We recognized a regional cohort of 180 patients with CH born between 1980 and 2018 in the catchment areas of Turku and Kuopio University Hospitals. Of these 180 subjects 60 (33%) answered to open question about the effects of CH on everyday life. When patients or their parents were queried whether CH affects their or their child’s life, 23.3% (14/60) answered affirmatively, mostly because of symptoms related to hypothyroidism.

The 15D questionnaire was sent to 101 patients and 29 of them (29%) completed it. Respectively, the 16D questionnaire was sent to 23 patients and 10 of them (43%) returned it. The patients who responded to the 15D questionnaire were between 16 and 40 years old (born between 1981 and 2005, median age 24.0 years). Respectively, the patients who responded to the 16D questionnaire were between 12 and 15 years old (born between 2005 and 2009, median age 14.6 years). Birth data and diagnostic characteristics at the time of diagnosis are described in Table 4, and there were no significant differences between participants and non-participants.

**Table 4:** Characteristics of the study populations in health-related quality of life research.

	15D group (*n* = 29)	Non-participants born at the same period (*n* = 72)	16D group (*n* = 10)	Non-participants born at the same period (*n* = 13)
Sex (male/female), *n*	11/18	28/44	3/7	6/7
Born from a multiple pregnancy, *n*	0	2	0	0
Parity (birth order), median (IQR)	2 (1–3)	2 (1–3)	2(1–2)	1 (1–2.5)
Mother’s age at birth, mean, years (95% CI)	28.9 (26.9–30.8)	28.7 (27.3–30.1)	28.7 (23.8–33.5)	28.9 (24.6–33.2)
Duration of pregnancy, median, days from due date (IQR)	4 (−4–12)	0 (−9.5–7.5)	0(−5.5–13)	8 (−3–13.5)
Apgar 1 min/5 min, median (IQR)	9 (8–9)/9 (8–9)	9 (8–9)/9 (9–9)	9 (8.5–9)/9 (9–9)	9 (8.5–9)/9 (9–9)
Birth weight, mean, SDS (95% CI)	0.1 (−0.4–0.6)	0.0 (−0.3–0.3)	0.1 (−0.6–0.9)	0.3 (−0.6–1.2)
TSH concentration at the birth, median, mU/l (IQR)	260 (135–400)	350 (180–475)	275 (171–423)	218 (100–295)
TSH concentration at the diagnosis, median, mU/l (IQR)	300 (200–383)	376 (182–510)	303 (123–418)	164 (54–301)
fT4 concentration at the diagnosis^a^, mean, pmol/l (95% CI)	8.2 (5.9–10.5)	9.2 (6.9–11.3)	10.1 (4.6–15.5)	11.8 (7.5–16.1)
Age at the onset of treatment, median, days (IQR)	4 (3–6)	4 (3–6)	3 (2.5–4)	4(3–4)
Initial dosing of levothyroxine, mean, mg/kg (95% CI)	8.0 (7.3–8.8)	7.8 (7.4–8.2)	9.9 (8.8–10.9)	9.9 (9.0–10.7)
Transient CH, *n*	2	1	0	0
TSH concentration during the study^b^, median, mU/l (IQR)	2.4 (1.6–5.2)	N/A	2.9(1.2–3.8)	N/A
fT4 concentration during the study^b^, mean, mU/l (95% CI)	20.6 (19.1–22.2)	N/A	19.5 (17.4–21.6)	N/A

*95% CI* 95% confidence interval, *IQR* interquartile range, *N/A* not applicable.

*P* value > 0.05 in all comparisons between participants and non-participants.

^a^Number of studied cases: 15D group *n* = 16, non-participants 15D *n* = 44, 16D group *n* = 7, non-participants 16D *n* = 11.

^b^Within 2 months of completing the questionnaire.

**Fig. 2:**
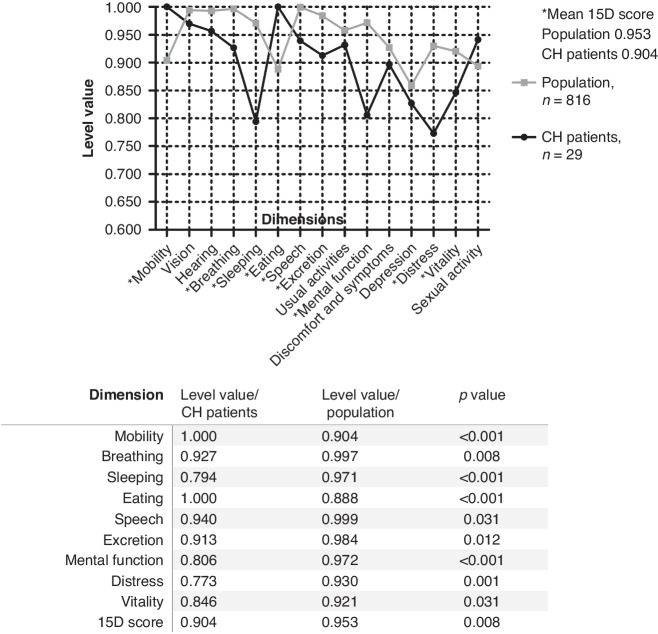
Self-reported health-related quality of life comparing 16- to 40-year-old patients with congenital hypothyroidism and general population using the 15D instrument. Statistically significant differences in mean values are marked with * and reported in the table under the curve. The difference in the mean 15D score is clinically important.

**Fig. 3:**
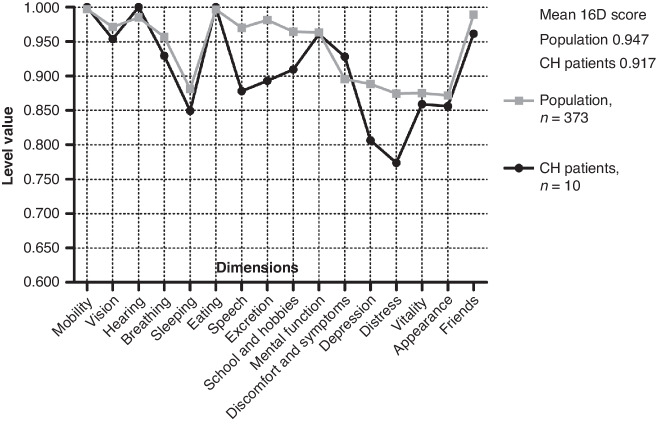
Self-reported health-related quality of life comparing patients 12- to 15-year-old patients with congenital hypothyroidism and general population using the 16D instrument. Differences in mean values are not statistically significant.

The mean 15D score was significantly lower in CH patients than in the age- and sex-standardized control population (0.904 vs. 0.953, *p* = 0.008), and this difference is also clinically important. CH patients reported significantly lower scores on the dimensions of breathing, sleeping, speech, excretion, mental function, distress, and vitality (Fig. 2). The mean 16D score was lower in CH patients but without statistical significance (Fig. 3).

To assess the possible effects of the initial TSH and fT4 concentrations and the age at the onset of the levothyroxine treatment, we divided the 15D group (adults) to subgroups based on these determinants. There was no difference in the mean HRQoL between patients whose levothyroxine treatment was started at the latest by the age of four days (*n* = 15) and those whose treatment began between the age of five and ten days (*n* = 14). Neither did the mean HRQoL differ between patients with an fT4 concentration of less than 5 pmol/l at diagnosis (*n* = 7) and patients with a higher fT4 concentration (*n* = 10), or between patients with a TSH concentration of less than 100 mU/l at the diagnosis (*n* = 6) and patients with a higher TSH concentration (*n* = 23).

## Discussion

Our study suggests that most patients with CH integrate well into society. The level of education as well as socioeconomic and marital status were similar in CH patients and their matched controls. Nevertheless, HRQoL was lower in CH patients compared to a sex- and age-standardized population and a remarkable proportion of CH patients experienced symptoms related to hypothyroidism.

Earlier studies have reported varied results on the course of life in CH patients. In a study of van der Sluijs et al.^15^, there was no difference between controls and young adult CH patients in marital status, educational level, need for special education or in living with parents. On the contrary, in a French study,^16^ CH patients were less likely to attain the highest level of education or the highest-intermediate socioeconomic category. They were also more likely to be outside the labor market and lived with their parents or without a partner. Another study of Léger et al.^26^ reported that school achievement was similar among patients with CH and in the national population. However, more severe forms of CH have been associated with a risk of not graduating from high school^16^ and with a risk of late entry into 6th grade.^26^

In the current study, the socioeconomic and marital statuses were similar in CH patients and their controls. This may reflect an improvement in the treatment, since patients in the previously mentioned studies were born between 1979 and 1988 and in current study between 1987 and 1992. In addition, cultural and social differences between countries may contribute to the different results.

Similar to the current study, previous European studies have also shown impaired HRQoL among children and young adults with CH.^15–17^ On the other hand, Sato et al. showed in a Japanese study that HRQoL of young adults did not differ from healthy controls.^14^ Van der Sluijs et al. reported that especially patients with severe CH are at risk for impaired HRQoL.^17^

It remains unclear, to which extent CH-related comorbidities affect patients’ everyday life. In the current study, HRQoL was lower on the dimensions of mental function and speech. In our previous study, the incidence of specific developmental disorders was higher in CH patients as compared to controls,^12^ which may affect the afore mentioned dimensions of quality of life. On the other hand, the mean value of the mobility dimension in the 15D instrument was not decreased, despite motor problems and delays in motor development in CH patients are widely reported.^11,27–29^ Another open question is the effect of a chronic disease per se on the quality of life with regular hospital visits, repeated laboratory tests and possible anxiety of parents.

The treatment of CH aims to normal development and growth by keeping thyroid hormone levels within the normal range. However, in the open question sent to the participants of the current study, a considerable number of patients or their parents (23.3%) reported that they experienced symptoms related to CH, which affect health or everyday life. In our recent study, parents reported significantly more symptoms of any kind during outpatient clinic visits when subnormal TSH values were measured, compared to visits with normal or supranormal TSH. Still, the distribution of symptoms related to over- or undertreatment was similar regardless of the TSH values.^20^ In the current study, there were no significant differences on the dimension of discomfort and symptoms in the 15D and 16D instruments between CH patients and a sex- and age-standardized population. Hence, it remains unanswered, how these experienced symptoms in the current study relate to thyroid hormone levels. However, this does not affect patients’ subjective experiences of their symptoms as being CH related.

The strength of this study is the versatile perspective to approach the social consequences of congenital hypothyroidism. A further strength was the parallel analysis of two cohort populations which allowed us to benefit from both nationwide registers with matched controls and a regional cohort which allowed us to study HRQoL. As compared to earlier studies, patients in the current study cohort were born between 1987 and 2009, and thus, received more modern treatment.

A notable limitation of the current study is the relatively low response rate to the HRQoL questionnaires. It is possible that patients experiencing symptoms or other problems related to CH were more likely to participate in the study. On the other hand, the patients with difficult challenges may not have been able to answer the questionnaires. However, these limitations do not affect the register data used in this study. Determinants influencing the quality and course of life warrant further investigation.

As van Vliet and Grosse^30^ stated earlier, newborn screening is necessary but not sufficient by itself to maximize the potential of people with CH. Careful monitoring of not only laboratory values and symptoms, but also cognitive, motor and behavioral development is important as well as ensuring adequate support. The importance of supporting and encouraging both the child and the parents at the time of diagnosis and during follow-up has been acknowledged earlier^31,32^ and is further supported by this study. In the current study, there was a trend of greater need for special education in patients with CH. It can be speculated if this extra support provided in schools contributes to equal success in adulthood.

## Conclusions

In this nationwide population-based study with matched controls, SES and marital status of CH patients did not differ from matched controls at the age of 25 years. Most CH patients integrate well into society, but the health-related quality of life is still impaired.

## Data Availability

The datasets generated and analyzed during the current study are not available due to data privacy and ethical and legal concerns.
